# Development of a complex intervention to improve participation of nursing home residents with joint contractures: a mixed-method study

**DOI:** 10.1186/s12877-018-0745-z

**Published:** 2018-02-28

**Authors:** Susanne Saal, Gabriele Meyer, Katrin Beutner, Hanna Klingshirn, Ralf Strobl, Eva Grill, Eva Mann, Sascha Köpke, Michel H. C. Bleijlevens, Gabriele Bartoszek, Anna-Janina Stephan, Julian Hirt, Martin Müller

**Affiliations:** 1 Institute of Health and Nursing Sciences, Medical Faculty, University of Halle-Wittenberg, Magdeburger Straße 8, 06112 Halle (Saale), Germany; 2 Institute for Medical Information Processing, Biometry and Epidemiology, Ludwig-Maximilians-University Munich, Marchioninistr. 15, 81377 Munich, Germany; 3 German Center for Vertigo and Balance Disorders, Ludwig-Maximilians-University Munich, Marchioninistr. 15, 81377 Munich, Germany; 4 Institute for General, Family and Preventive Medicine, Paracelsus Medical University, Strubergasse 21, 5020 Salzburg, Austria; 5 Institute of Social Medicine and Epidemiology, Nursing research group, University of Lübeck, Ratzeburger Allee 160, 23538 Lübeck, Germany; 6 CAPHRI Care and Public Health Research Institute, Department of Health Services Research, Living Lab on Ageing and Long-Term Care, Maastricht University, Universiteitssingel 40, 6229 ER Maastricht, The Netherlands; 7 University of Applied Sciences for Social Work, Education and Nursing, Dürerstraße 25, 01307 Dresden, Germany; 8 Faculty of Applied Health and Social Sciences, Rosenheim University of Applied Sciences, Hochschulstraße 1, 83024 Rosenheim, Germany

**Keywords:** Contractures, Nursing homes, Social participation, International classification of functioning, Disability and health (ICF), Complex intervention, Quality of life

## Abstract

**Background:**

Joint contractures in nursing home residents limit the capacity to perform daily activities and restrict social participation. The purpose of this study was to develop a complex intervention to improve participation in nursing home residents with joint contractures.

**Methods:**

The development followed the UK Medical Research Council framework using a mixed-methods design with re-analysis of existing interview data using a graphic modelling approach, group discussions with nursing home residents, systematic review of intervention studies, structured 2-day workshop with experts in geriatric, nursing, and rehabilitation, and group discussion with professionals in nursing homes.

**Results:**

Graphic modelling identified restrictions in the use of transportation, walking within buildings, memory functions, and using the hands and arms as the central target points for the intervention. Seven group discussions with 33 residents revealed various aspects related to functioning and disability according the International Classification of Functioning, Disability and Health domains body functions, body structures, activities and participation, environmental factors, and personal factors. The systematic review included 17 studies with 992 participants: 16 randomised controlled trials and one controlled trial. The findings could not demonstrate any evidence in favour of an intervention. The structured 2-day expert workshop resulted in a variety of potential intervention components and implementation strategies. The group discussion with the professionals in nursing homes verified the feasibility of the components and the overall concept. The resulting intervention, Participation Enabling CAre in Nursing (PECAN), will be implemented during a 1-day workshop for nurses, a mentoring approach, and supportive material. The intervention addresses nurses and other staff, residents, their informal caregivers, therapists, and general practitioners.

**Conclusions:**

In view of the absence of any robust evidence, the decision to use mixed methods and to closely involve both health professionals and residents proved to be an appropriate means to develop a complex intervention to improve participation of and quality of life in nursing home residents. We will now evaluate the PECAN intervention for its impact and feasibility in a pilot study in preparation for an evaluation of its effectiveness in a definitive trial.

**Trial registration:**

German clinical trials register, reference number DRKS00010037 (12 February 2016).

## Background

Joint contractures are characterized by restrictions in physiological joint mobility and can even result in immobility [1]. Joint contractures have a wide range of causes, including immobility, pain, and neurological conditions [2–5]. Not surprisingly, joint contractures are a common problem among older, frail people living in nursing homes [6, 7] and greatly affect not only the capacity to perform daily activities (such as toileting, walking) or to participate in social life but also the need for nursing care [6, 8–10]. Studies have shown that participation restrictions are most relevant from the perspectives of both the affected individuals and the health professionals involved in their management and care [10–12].

Interventions that target the broader goal of improving social participation in nursing home residents with joint contractures face several challenges. According to the WHO’ model of the International Classification od Functioning, Disability and Health participation restrictions are problems an individual may experience in involvement in life situations [13]. First, the population shows great clinical variation and includes both frail but ambulatory individuals and individuals who are already heavily restricted in their mobility or are even bedridden. Second, persons with joint contractures can have varying preferences regarding their social participation. Third, some individuals may already have one or several joint contractures, whereas others are at risk of developing joint contractures. In addition, because multimorbid residents with joint contractures might be cared for by many different individuals, a successful intervention should address all professionals in nursing homes, including qualified nurses and assistant staff, therapists, and physicians, as well as informal caregivers. With these challenges in mind, it is clear that a successful intervention aimed at improving participation in nursing home residents with joint contractures must by its very nature be complex. Careful development of such a complex intervention must consider both theoretical findings and empirically identified influencing factors.

Our aim was to develop a complex intervention to improve participation in nursing home residents with joint contractures that systematically integrates evidence and account for the perspectives of all stakeholders [14].

## Methods

The development approach followed the UK MRC framework [15], the most widely used guidance for the development of nursing interventions [16]. The MRC framework proposes a four-phase approach to develop and evaluate complex interventions. This paper comprises all aspects of the development phase, including exploration of relevant theories, identification of the existing evidence, exploration of potential intervention components, modelling of the intervention components, and the implementation process. The study combines qualitative and quantitative methods in a mixed-methods design. To describe the development process in detail, we adhered to the criteria for reporting the development and evaluation of complex interventions in health care [17].

**Fig. 1:**
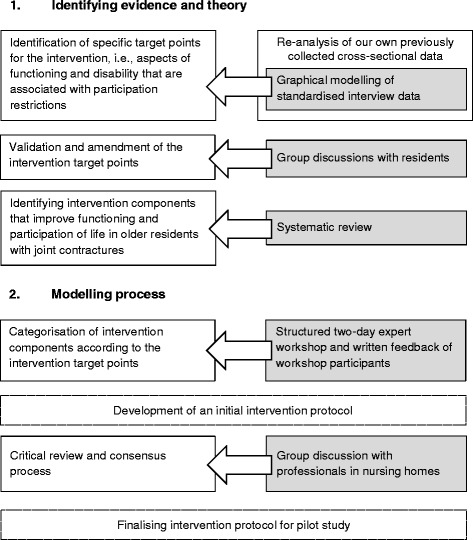
Overview of the intervention development process

An overview of the intervention development process is presented in Fig. 1.

### Identifying evidence and theory

We had previously conducted standardized [10, 11] and qualitative interviews [18] with nursing home residents and patients in geriatric rehabilitation hospitals. Our purpose was to assess and describe the prevalence of activity limitations and participation restrictions of older persons with joint contractures, and the impact of joint contractures on functioning and social participation from the patients’ perspective. In addition, we explored the problems older people with joint contractures experience by conducting an Internet-based expert Delphi survey with international health professionals [12]. As a result of our preparatory studies, improvement of social participation and quality of life emerged as the primary objectives of our intervention, with emphasis on the role of contextual factors in participation and quality of life of nursing home residents with joint contractures.

As in the preparatory studies, we used the biopsychosocial model of the International Classification of Functioning, Disability and Health (ICF) of the World Health Organization (WHO) to guide the theoretical development of the intervention, especially to model potential interactions of the intervention components with the targeted outcomes. The ICF model can be understood as the operationalization of functioning and health as the outcome of the dynamic interaction between a person’s health condition and his or her personal and environmental contextual factors [13].

For this study, we explored the theoretical underpinnings and the available evidence base using a stepwise approach (Fig. 1).

#### Graphical modelling of standardized interview data

To investigate potential intervention goals, we analysed data from our previous cross-sectional study by means of graphical modelling [10, 11] Graphical modelling is an approach to visualize conditional dependencies between various variables where most relevant dependencies are displayed in a netlike structure by drawing a graph. The associations within graphical models are estimated using generalized linear regression analysis [19–21]. We assumed that variables that are associated with multiple other variables as displayed in the graphs are valuable starting points for interventions. The cross-sectional study was conducted between February and July 2013 in three acute-geriatric hospitals in and around Munich, Bavaria (Germany) and in eleven nursing homes and three geriatric rehabilitation hospitals in and around Witten, North Rhine–Westphalia (Germany). Two hundred ninety-four participants 65 years of age or older with at least one diagnosis of joint contracture were interviewed face-to-face via a standardized questionnaire. The study determined the extent of limitations and restrictions of functioning related to joint contracture in older persons in geriatric care.

#### Group discussions with nursing home residents

To validate the findings from the graphical modelling, moderated group discussions with nursing home residents were carried out in nursing homes in two areas in Germany, Munich (Bavaria) and Witten (North Rhine–Westphalia), between March and June 2015. Two of the authors (GB, AS) used an interview guide that was developed to identify barriers and facilitators for activities and participation and to validate the intervention goals identified by graphical modelling. Before the start of the focus group meeting, we asked participants to complete a short questionnaire on their demographic characteristics, location of the joint contracture, and current care level and to classify their functioning using a visual analogue scale. Each group consisted of four to five nursing home residents selected according to predefined inclusion criteria and asked by the nursing home managers to participate. The inclusion criteria were (1) an age of 65 years or above with at least one diagnosis of joint contracture, (2) the ability to give informed consent for themselves, and (3) the cognitive ability to participate in and follow a group discussion, judged by an expert opinion of a nurse in charge. The sample size was determined by data saturation––i.e., the point at which an investigator has obtained sufficient information from the field [22]. A signed informed-consent form was obtained from each participant before the study began. One researcher moderated the group discussion interviews, and two persons recorded the minutes. To avoid a formal interview situation and foster a friendly and open-minded conversation, no audio recordings were collected. Two researchers (AS, JH) analysed the minutes independently using the meaning condensation procedure [23]––a qualitative content analysis approach––together with the ICF linking procedure, a method that utilizes the ICF as a fixed-category system [24]. The two researchers’ versions were merged, and differences were discussed with support from a senior researcher (MM). All analyses were carried out in Microsoft Excel.

#### Systematic review

To identify potential intervention components for prevention and treatment of disability due to acquired joint contractures in older people and to determine positive and adverse effects of interventions, a systematic review was conducted (latest search August 2016). The full report can be found elsewhere [25]. In brief, the databases Cochrane Library, PubMed, EMBASE, PEDro, CINAHL, trial registries, reference lists of retrieved articles, and scientific congress pamphlets were systematically searched, including the following combined search terms, among others: contracture [MeSH], joint contracture, social participation, aged [MeSH], randomized controlled trial, controlled clinical trial. Controlled and randomized controlled trials in English or German that compared an intervention with another intervention or standard care were included. Critical appraisal followed the Cochrane Handbook for Systematic Reviews of Interventions, version 5.1.0 [26]. Two researchers independently selected studies for inclusion/exclusion, assessed the methodological quality trials, and extracted data.

### Modelling process

#### Structured expert workshop

In a 2-day workshop with a structured consensus process, geriatricians and experts in nursing and rehabilitation science identified relevant intervention components. After presentation and discussion of the findings from the first part of the study, experts collected ideas for potential interventions and discussed factors that might influence the intervention components and successful implementation. Methods used to structure and promote the discussion process included brainstorming, plenary discussion, group work, and the development and presentation of a poster. All proposed intervention components were evaluated regarding their ability to improve the residents’ participation against the background of the ICF model.

#### Written feedback of workshop participants

After the workshop, the study team summarized and detailed the results of the workshop and asked the participants to give written feedback via e-mail. The experts were asked to amend missing information on the topics for which they were responsible during the workshop and to provide additional feedback on all other components. Disagreements were resolved in an iterative discussion via e-mail.

After completion of the feedback process, the research team prioritized the intervention components according to their assumed feasibility. Next, an implementation approach on the revised intervention components was developed. The initial intervention protocol was validated by five participants in the expert workshop. The implementation approach is based on the theory of planned behaviour [27] and uses nominated key nurses as multipliers, who act as a change agent in the nursing home. The appropriateness of this approach has been proven [28].

#### Group discussion with professionals in nursing homes

In a moderated group discussion, nursing professionals in North Rhine–Westphalia with experience in innovative change processes gave feedback on the intervention protocol regarding the interventions’ relevance, comprehensiveness, and feasibility and on barriers that could be expected during the implementation. A member of the research team (GB) moderated the discussion using a structured interview guide, and a research assistant documented the interview in written form. This documentation was validated by the participants of the group discussion. Finally, in a telephone conference, all members of the research team discussed the intervention protocol and agreed on its final version.

## Results

### Graphical modelling

Standardized interview data from 294 persons were reanalysed. The participants’ mean age was 80.4 years (range, 65.0 to 99.7 years; SD, 7.54 years); 195 participants (66%) received care in geriatric rehabilitation facilities and 99 (34%) in nursing homes; 198 (67%) were female. The graphic model revealed that restrictions in the use of transportation, walking within buildings, memory functions, and using hands and arms had the greatest association with other restrictions and might therefore be promising target points for the intervention.

### Group discussions with nursing home residents

Seven group discussions (5 in Munich and 2 in Witten) were conducted with 33 nursing home residents with joint contractures (88% female; mean age, 85 years; SD, 6.99 years); 61% had joint contractures in the upper and the lower extremities, 15% solely in the upper extremities, and 24% in the lower extremities. The participants’ characteristics are presented in Table 1. The interviews averaged 45 min (range, 30 to 60 min).

**Table 1:** Characteristics of residents in the group discussion (*n* = 33)

Variables		
Age in years, mean (SD)	84.6	(7.0)
Female gender, *n* (%)	29	(88)
Self-rated functioning^a^, mean (SD)	4.72	(1.9)
Localization of joint contracture, *n* (%)		
Lower extremity	8	(24)
Upper extremity	5	(15)
Lower and upper extremity	20	(61)
Level of care dependency^b^, *n* (%)		
Minor	6	(18)
Considerable	15	(45)
Severe	10	(30)
Most severe	0	(0)

^a^Visual analogue scale, range 0 to 10 = sad face to smiling. Data not available for three participants

^b^For description of the functional and cognitive status, we used levels of care dependency as assessed by expert raters of the medical service of the German statutory health insurance system (0 = minor, 1 = considerable, 2 = severe, 3 = most severe). Data not available for two participants

Restrictions in the ICF categories *Mobility and Self-care* and problems in the ICF domain “Environmental factors” were most often reported by nursing home residents with joint contractures. The reported ICF domains and categories are displayed in Table 2.

**Table 2:** ICF domains and categories from group discussions with 33 nursing home residents

ICF domains and categories	
Body functions	
Mental functions	
Sensory functions and pain	
Genitourinary and reproductive functions	
Neuromusculoskeletal and movement-related functions	
Body structures	
“General physical decline”	
Activities and participation	
General tasks and demands	
Major life areas	
Community, social, and civic life	
Domestic life	
Interpersonal interactions and relationships	
Communication	
Mobility	
Self-care	
Environmental factors	
Products and technology	
Service, systems, and policies	
Attitudes	
Support and relationships	
Natural environment and human-made changes to environment	
Personal factors	

### Systematic review

Seventeen studies with 992 participants met the inclusion criteria: 16 randomised controlled trials and one controlled trial (four in nursing homes, 13 in the community). Four studies reported on splints, nine on stretching exercises, and one each on ultrasound, passive movement therapy, a bed-positioning program, and a group exercise program. The methodological quality of the studies varied. Five of seven studies that assessed active stretching programs for healthy older people reported statistically significant effects on joint mobility in favour of the intervention. One of four studies that investigated the effects of splinting reported significant improvement of the passive range of motion. One study of a group exercise program observed significant improvements in activities. No positive effects were reported for active stretching programs for frail older people, ultrasound, passive movement therapy, and a bed-positioning program. Studies rarely assessed pain, quality of life, activity limitations, and participation restrictions. Overall quality of evidence was low and therefore not a reliable basis for further development. Detailed findings appear elsewhere [25].

### Structured expert workshop and written feedback of workshop participants

The two-day expert workshop with eight participants (two experts of geriatric sciences, three experts of nursing sciences, and three experts of rehabilitation sciences) and the subsequent written feedback resulted in a variety of potential intervention components, such as useful assessments and measures to reduce environmental barriers, strategies to improve interprofessional care, and strategies to consider personal factors in promoting mobility and to engage residents in social activities. Several implementation strategies also identified were qualification of multipliers, peer mentoring of multipliers, qualification of the nursing home staff, and strategies to involve nursing home managers, social workers, informal caregivers, and therapists in change processes.

The research team prioritized suggestions regarding the intervention components according to the anticipated feasibility in the nursing home setting. The team developed a delivery approach for the revised intervention components according to the suggestions by the experts, and five participants of the expert workshop validated both the delivery approach and the revised intervention protocol.

### Group discussion with professionals in nursing homes

**Fig. 2:**
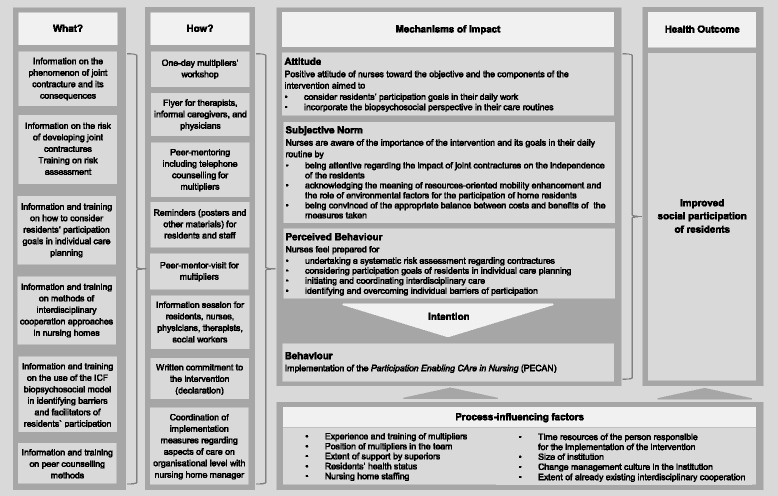
Logic model of the Participation Enabling CAre in Nursing

We discussed the pre–final intervention protocol with four nursing professionals: a skilled nurse responsible for admission processes acting as a multiplier of nursing guidelines to support mobility, a head of nursing, a nursing home manager, and a skilled nurse responsible for quality management. The participants recommended an intensive collaboration of nurses with social workers and nursing assistants for social care in the nursing homes. They also highlighted the necessity to plan for sufficient time between each implementation step to allow the multipliers to deal with their regular tasks in addition to their new roles. The participants judged the implementation approach as feasible and comprehensive and also considered the content of the workshop to be relevant and consistent. All discussed checklists and tools received confirmation of their usefulness and focus, except that participants did not consider that a developed guideline about goal setting in nursing plans was feasible. The logic model (Fig. 2) displays the final version of the complex intervention named Participation Enabling CAre in Nursing (PECAN).

### PECAN intervention

The PECAN intervention is a multifactorial program to improve care of nursing home residents with joint contractures. The policy is to improve residents’ social participation through reduction of hindering environmental factors, facilitation of personal factors, and support of mobility. Because nursing homes use a wide range of documentation formats, as well as different risk assessments and planning tools, the PECAN intervention does not aim to implement additional measures or assessments into standard care. The intervention enables nurses to critically review organizational procedures and residents’ care plans according to predefined criteria, to initiate changes into daily care, and to prepare themselves to act as change agents of the nursing home’s daily routines.

#### Kick-off meeting with the head of nursing/nursing home manager

In a first meeting with the head of nursing and the nursing home manager, the policy of the PECAN will be discussed and a declaration must be signed to formally document and reinforce the institutional commitment. The declaration will be placed in full view of all visitors.

#### Multipliers’ workshop

The key component of the intervention is a 1-day workshop for nurses, who are nominated as multipliers of the intervention in the nursing homes to offer education and counselling to their colleagues.

The workshop for nominated nurses comprises the following activities:

- Sharing of information about the causes, consequences, and risks of joint contractures;
- Critical review of risk assessments used in the nursing home;
- Training in ways to consider residents’ participation goals in the individual care planning through presentation of case vignettes and case reports;
- Presentation of information on methods of interdisciplinary collaboration;
- Training in the use of the ICF biopsychosocial model to identify barriers and facilitators of residents’ participation;
- Provision of information on measures to prevent and treat joint contractures and their suitability for residents with different mobility restrictions;
- Training in peer counselling methods.

#### Information session

The researchers developed an information session for residents, informal caregivers, and staff of nursing homes to inform everyone about causes, risks, and consequences of joint contractures, to describe the model of the ICF and the PECAN intervention, and to introduce the implementation approach, the multipliers, and their tasks.

#### Peer-mentoring

The implementation process includes a mentoring approach, in which the multipliers receive counselling by a nurse of the research team (the mentor) on a regular basis to support role finding and planning of the implementation. The mentoring approach is derived from a peer assistance and review process that has already been proven successful in other circumstances [29]. At the beginning of the mentoring process, the multipliers receive counselling and support to determine implementation measures during a peer-mentor visit in the nursing home by an interdisciplinary team: an external peer experienced in change management in nursing homes, a therapist, and the mentor. During this visit, the multipliers critically review organizational procedures to identify barriers and facilitators of implementation using a checklist with predefined criteria. The required changes on an organizational level will be planned together with the head nurse, supported by the mentor. Moreover, the interdisciplinary team critically reviews individual care plans using a structured assessment tool to identify barriers and facilitators of PECAN and will plan changes in care with counsel by the external peer experts.

The multipliers will receive counselling by their mentor via phone calls every second week throughout the first two months of implementation. Thereafter, telephone calls will be held upon request, at least once a month. Multipliers are expected to train their colleagues in procedures of the PECAN intervention.

#### Supportive materials

A further component of the intervention, the use of posters and other written material, is intended to remind residents and staff. The written material comprises leaflets offering information about the intervention and contact details of the multipliers and the study team to be provided for external therapists and physicians, as well as informal caregivers.

**Fig. 3:**
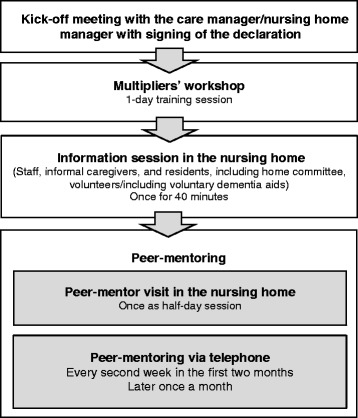
Overview of the implementation approach

Figure 3 presents the implementation approach of our intervention PECAN.

## Discussion

We describe here the development of a theoretically and empirically informed complex nursing intervention aimed at improving social participation and quality of life in nursing home residents with joint contractures. The intervention is now ready for implementation within a pilot study.

Our intervention is based on findings from the literature and on the experiences of nursing home residents, managers of nursing homes, geriatricians, and nursing and rehabilitation scientists.

Whereas the graphical modelling and the group discussions with the nursing home residents revealed meaningful target points of the intervention, the systematic review did not contribute to the development. This review [25] revealed a lack of studies relevant for nursing home residents with joint contractures, and the few existing studies did not show sufficient effects of interventions. The findings from the interviews with nursing home residents underscored that immobility alone does not lead to restrictions in participation, but these restrictions are also influenced by a range of environmental and personal factors. Based on this information, we derived intervention goals that guided the development of the intervention components.

As a result of this modelling process, we developed a qualification scheme for nurses and an approach to support transfer into daily routine for the implementation of the intervention.

According to the biopsychosocial model of the ICF, participation restrictions are associated with impairment in body functions and structures and might be facilitated or hindered by environmental and personal factors. As such, the focus of our intervention is to reduce hindering, strengthen supportive environmental factors, and facilitate positive personal factors, such as the residents’ motivation to maintain mobility and to engage in social activities within their current living situation [13]. Support of mobility is a key aspect of our intervention because of the relationship between immobility and joint contractures. Several studies suggest the positive effects of promoting physical activity on physical functioning in residents of nursing homes [30]. In this regard, our intervention is in line with other mobility programs like function-focused care [31, 32]. Our intervention uses the same strategies to promote physical activities that were successfully applied in the function-focused care concept, such as education, environmental assessment, goal setting, and mentoring. However, our intervention approach is novel, in that it expands its focus on participation and associated factors and therefore adds a range of possible interventions.

To implement the intervention, we chose a multiplier approach, which is a proven strategy for implementation of changes of nursing home care [28, 33–35]. This approach is accompanied by varying strategies to address all persons who are relevant to the improvement of residents’ participation. Our assumptions about meaningful intervention components (as described in the logic model, Fig. 2) were driven by facilitators of implementation identified in previous research steps. This is comparable to other complex interventions in geriatric settings [36].

Our study uses the UK MRC framework [15] for development and evaluation of complex interventions, which has demonstrated its usefulness. Due to the weakness of the evidence that could have informed the intervention development process, we involved key stakeholders at different stages of intervention development to keep a broad and well-informed perspective.

The involvement of residents in the modelling process aimed at identifying participation priorities and barriers to participation and individual problem-solving strategies. However, the feedback from the residents added less information than expected and suggested that frail older people are likely to adapt to their physical disability and thus to their expectations on participation [37]. To overcome this unwanted phenomenon, strategies are needed enhancing older people’s sense of self-worth and helping them understand the way how their social participation can be facilitated [38]. It has to be taken into account that residents with severe cognitive decline were not part of the group discussion as well as the other research steps did not focus on the specific needs of residents with severe cognitive decline. Hence, the intervention might not be applicable to this group of residents.

Consultation with experts proved to be a helpful approach to support the definition of intervention goals and collection of ideas about intervention components and possible implementation approaches. However, the information generated by the experts ultimately required further synthesizing efforts by the research team using iterative consensus rounds. In addition, facilitation of the process had to be stringent to keep participants on track, especially regarding the empirically generated intervention goals.

Because the UK MRC framework does not explicitly discriminate between what should be implemented and how it should it be implemented, the logic model [39, 40] helps to describe how the intervention might work and to differentiate between intervention content (“what”) and implementation components (“how”).

The intervention development was clearly theory-driven, using the ICF model in the graphic modelling process, in analysing the data on group discussions with residents, and in informing the intervention modelling process. The theory of planned behaviour worked well in elaborating the implementation components.

## Conclusions

The PECAN intervention is ready for a pilot study investigating its impact and feasibility. A necessary adjunct to the pilot study will be a comprehensive process evaluation to identify the relevant elements of the intervention and to explore the barriers and facilitators of a successful implementation approach. Although the intervention was developed for nursing home residents with joint contractures, residents at risk of developing joint contracture might also benefit from the PECAN intervention. This question might be answered in a subsequent implementation study.

Our methodological approach might serve as a template for structured intervention development processes in areas where the evidence base is weak.

## Abbreviations

ICF: International Classification of Functioning, Disability and Health of the World Health Organization
MRC: Medical Research Council
PECAN: Participation Enabling CAre in Nursing
SD: Standard deviation
